# Meetings of Societies

**Published:** 1902-03

**Authors:** 


### MEETINGS OF SOCIETIES.

### Bristol Medico-Chirurgical Society.

*December 11th, 1901.*

Dr. Barclay J. Baron, President, in the Chair.

Dr. J. O. Symes showed microscopic preparations of the blood from a patient suffering from **malignant (æstivo-autumnal) tertian fever,** who had been under the care of Dr. Skerritt. Crescentic and ovoid bodies were numerous, the smaller intra-corpuscular plasmodia being rare.—Dr. Newman Neild showed specimens of the **malarial parasite obtained from a case of tertian ague.** They illustrated two stages in the development of the organism.

Dr. Michell Clarke showed three patients. The first was a man, aged 42, with **amyotrophic lateral sclerosis.** He had had syphilis eight years ago, which had been thoroughly treated, but no other illness. The disease had begun nine months previously with the development of weakness in the left shoulder and dull pains there, and weakness in the neck muscles. For five months he had also had weakness of the legs. At the present time there was wasting of the following muscles: both great pectorals, but especially the left; the left deltoid and biceps; the trapezii in their lower two-thirds; and the supra- and infra-spinati. In all wasting was mostly on the left side. Slighter wasting was also obvious in the upper part of the left trapezius and the small muscles of the right thumb. The faradic reactions were lessened and the galvanic increased in all the wasted muscles, and the reaction of degeneration was yielded by the muscles which were most wasted, and fibrillary tremors were noticed in them. The knee-jerks were exaggerated, and Babinski's sign was present. The patient was improving under treatment with rest, galvanism, and hypodermic injections of strychnine. The second patient, a man of 61, was suffering from a **vascular lesion of the pons (hemorrhagic).** Two years ago he had had paralysis of the right third nerve, from which he had recovered. Four weeks previously he fell down, vomited, and had vertigo, and could not raise himself. This attack left him with absolute loss of power of swallowing; right hemianæsthesia, which was partial only, pain being chiefly affected, and was limited by the middle line; paresis of the left sixth nerve with diplopia; slight left ptosis; paresis of the left fifth nerve in its motor division; loss of taste in the anterior two-thirds of the tongue on the right side; paresis of the left seventh nerve and of the right vocal cord; and absence of the right knee-jerk. The pupils, tongue, and the senses of sight, smell, and hearing were normal. The patient had regained the power of swallowing in three weeks, and recovered the lost knee-jerk and from his loss of sensation. The third case, a man of 26, had **hereditary or family cerebellar ataxy.** The symptoms had been present six months. The patient's brother had been affected with similar symptoms for one year. Dr. Clarke also showed a specimen of **necrosis and exfoliation of the arytenoid cartilages,** which had followed enteric fever.—Mr. C. H. Walker said that he had seen the second patient at the Eye Hospital, when he had paralysis of the right third nerve. When the patient had almost recovered from that, under iodide of potassium, paresis of the left sixth nerve had developed.—Dr. F. H. Edgeworth mentioned the case of a patient who developed right hemiplegia three months after getting syphilis. In spite of mercury, three weeks later left hemiplegia developed, and then swallowing became impossible, though it had been unaffected by the first attack.—Dr. Michell Clarke, in replying, said that most physicians differentiated progressive muscular atrophy from amyotrophic lateral sclerosis, and considered the former a disease confined to the lower neurons, whereas in the latter both upper and lower neurons were affected.

Mr. J. Paul Bush, C.M.G., read a paper on **a case of gastro-jejunostomy** (see p. 57).—Dr. Shaw, under whose care the patient had been in the first instance, commented upon the satisfactory immediate results of the operation, and alluded to the difficulty of early diagnosis in this disease, and to the fact that patients were often unwilling to undergo the operation. —Mr. C. A. Morton favoured the posterior method of gastro-jejunostomy. He thought that in the anterior method the functions of the jejunum were more in danger of being interfered with by distension of the colon, than were the functions of the colon by the pressure of the displaced coil of jejunum. He had not found the posterior method more difficult than the anterior, as it was alleged to be. In one case he had used Mayo Robson's bone-bobbin, which he was getting to like for this and other operations. The jejunum in the operation naturally came into such a position that its peristalsis was in the same direction as that of the stomach, and it would be more difficult to fix it so as to reverse the direction of the peristalsis. —Mr. R. G. P. Lansdown alluded to a case in which he had performed the operation by the anterior method with very satisfactory results. The stomach in that case had been so fixed that the posterior method was impracticable. The patient, like Mr. Bush's, had been remarkably benefited for six months, and had only begun to lose ground again during the last few days.—Dr. Michell Clarke pointed out that even if physicians were able to make an early diagnosis they would not always get the opportunity, because patients would still often come for treatment when the disease was advanced.—Dr. Groves cited the results Mayo Robson had obtained in the operation. The most brilliant results had been in cases of gastric ulcer. Some cases which were supposed to be malignant had proved at the operation not to be malignant, and had been permanently benefited, which was a point in favour of operating. The posterior operation was more difficult to perform on the cadaver than the anterior.

Mr. A. W. Prichard read notes on **a case of acute intussusception.** The patient was a very young child. At the time of operation, about six hours after the onset of the symptoms, the pulse was uncountable. There was resistance in the left flank and a rectal swelling palpable. Median laparotomy was performed and the intussusception reduced. On the second day there was some collapse. A good recovery ensued and the patient was shown at the meeting.—Mr. J. Lacy Firth alluded to three cases of acute intussusception in very young children for which he had opened the abdomen. The patients had recovered. The third of these patients was in a desperate state at the time of the operation, with fæcal vomiting, a wretched pulse, and protrusion of an ileo-cæcal intussusception three inches beyond the anus. He thought it was better on the whole, even when the symptoms were mild, to perform laparotomy for intussusception as soon as the diagnosis was made, rather than to try inflation.—Mr. Paul Bush preferred to treat these cases by laparotomy. The injection treatment often failed to reduce the intussusception completely, and owing to the possibility of rupturing the bowel, laparotomy was really a safer procedure.

Mr. F. H. Rose read a paper on **ethyl chloride as a general anæsthetic** (*vide* p. 53).—Dr. J. O. Symes was not convinced that ethyl chloride yielded any better results than nitrous oxide gas. It seemed to take a little longer to anæsthetize the patient than with gas. The period of excitement which it produced, the occasional vomiting, and the fact that there had been a death under it in the first 2,000 administrations were points against it. Anæsthesia lasting a minute and a half was usually enough for a throat operation, but fifty seconds seemed as much as ethyl chloride could be relied upon to give.—Dr. Elwin Harris considered that it often failed to produce anæsthesia. The patients often yelled loudly during the administration. There was much want of uniformity in its action. On one or two occasions the patients had become dusky. He did not think that this anæsthetic could be recommended at present.—Mr. F. H. Rose in reply said that sometimes he had anæsthetized the patient in thirty seconds, at other times it had taken from five to six minutes. Some patients had not struggled, others had. The duration of anæsthesia was distinctly longer than that under nitrous oxide gas.

---

*January 8th, 1902.*

Dr. Barclay J. Baron, President, in the Chair.

Mr. C. King Rudge was elected a member of the Library Committee in the place of Mr. L. M. Griffiths, resigned.

Mr. Dobson proposed that the Society should give a special vote of thanks to the retiring librarian, Mr. L. M. Griffiths. For eleven years Mr. Griffiths had devoted a vast amount of time and labour to the library, and he had been a member of the Committee of the Society from its foundation. The state of the library sufficiently demonstrated the great industry of the late librarian.

Dr. Shingleton Smith seconded the resolution, and expressed admiration for Mr. Griffiths' work, and regret that it was now necessary to accept his resignation.

The resolution was cordially carried.

Mr. F. W. Crossman showed a **patient with a loud aortic murmur.** The patient's brother had been attended for a similar affection. Mr. Crossman thought the cause was probably the same in each case; viz., a partial rupture of the valve from a sudden strain.—Dr. Theodore Fisher thought musical murmurs were usually mitral, and asked what was the evidence of this one being aortic.—Dr. Watson Williams pointed out that the second aortic sound was lost and replaced by the bruit in this case.—Dr. Cave mentioned a case in which a similar murmur developed after a strain from lifting a piano. In that case the murmur was audible two feet away from the patient.—Dr. Stack thought that mitral bruits were not audible away from the body.—Dr. Michell Clark said that the murmurs heard at a distance were not always the loudest, they were often of medium intensity. He alluded to the audibility of a tapping sound which could be heard with each heart beat in some aortic cases by auscultation over arteries; often in the dorsalis pedis even.—Dr. Harrison thought it was the musical as distinguished from the noisy quality of a murmur which rendered it audible away from the body.—Dr. Newman Neild mentioned a case of phthisis in which a cardio-pulmonary bruit had been audible at a distance of five inches from the patient. It had been distinct when the patient was standing, but absent when he was lying down.

Mr. G. Munro Smith showed the following cases and specimens: (1) **A patient from whom he had removed practically the entire clavicle,** which had necrosed. In spite of the absence of the bone, which had not re-formed, the patient could move the arm on the affected side as strongly and completely as on the sound side. (2) **Two large renal calculi,** weighing together 462 grains, which he had successfully removed from a kidney. Twenty-seven minute calculi were passed at intervals in the three weeks immediately following the operation. (3) A specimen of **tonsillar calculus.** (4) **A concretion from the intestines of a horse** which had caused the animal's death. Though so stony in appearance the microscope showed it was entirely of vegetable structure.—Mr. Hancocke Wathen showed several **enteroliths obtained from horses,** and discussed their causation.—Dr. Kenneth Wills mentioned a tonsillar calculus he had seen which a patient had coughed up. Its nucleus was a medicinal pill.

Dr. Stack showed a specimen of **a ruptured intracranial carotid aneurysm** from a case of Dr. Shaw's. The patient, aged 22, had a basal cardiac bruit, slight displacement outwards of the cardiac apex, and double optic neuritis with much swelling of the disc. The diagnosis of a cerebral tumour with hemorrhage into it had been entertained. No cause for the aneurysm had been discovered. There was no renal disease or endocarditis.

Dr. Theodore Fisher showed a specimen of **acute tuberculosis of the lung**, obtained from a patient whose abdomen showed abundant intestinal adhesions, the result of old and obsolete tubercular peritonitis.

Dr. F. H. Edgeworth read a paper on **Transient Renal Hæmaturia** due to chill. Notes were given of three cases. In each after a definite exposure to cold the urine became deeply blood-stained, and blood casts were abundant. On the second day the blood and urine diminished in quantity. In one of the cases at least recurrence of similar attacks had been noticed. It was important to consider the relation of the affection to acute Bright's disease. The short duration, the complete and rapid recovery, and the recurrence of this affection distinguished it from Bright's disease.—Dr. Shingleton Smith thought the disease was really acute Bright's disease, in which the early and free hemorrhage favourably influenced the course and duration of that affection.—Mr. Roger Williams alluded to the advances which had been made in the diagnosis of renal affections, for he remembered the time when renal calculus and tubercular disease of the kidney were rarely diagnosed. The explanation of some cases of hemorrhage from the kidney was to be found in the existence of villous growths in the renal pelvis or ureter.—Dr. Michell Clarke spoke of the great utility of the electrical centrifuge in examining the urine for casts. Without the use of this instrument existing evidences of Bright's disease might easily be overlooked.—Dr. Edgeworth could hardly believe that Bright's disease could frequently recur, leaving the kidney quite healthy in the intervals, as happened in some of these cases.

Dr. J. Odery Symes read a paper on "**some urinary infections, with special reference to their treatment by urotropine**" (*vide* p. 27).—Mr. Carwardine alluded to the difficulty of obtaining urine from the bladder without contaminating it. He had found urotropine more effective in the cases in which the urine was alkaline. It made such urine acid. He had used it as a prophylactic against urethral fever. If formaline was liberated in the nascent state in the urine by the decomposition of urotropine, it would be more powerful even than usually.—Dr. Fisher stated that in a research at the Johns Hopkins University pyuria had been found to exist in ten per cent. of a number of cases of typhoid fever, and this percentage corresponded with the percentage of typhoid cases in which Dr. Symes had found bacilli in the urine.—Dr. Neild emphasized the importance of a dose of the drug being taken the last thing at night, owing to the fact that it was rapidly excreted. Phosphate crystals in acid urine were found after the drug had been given.—Dr. Cave mentioned that he had twice seen hæmaturia produced by the drug, and others had reported it. In one of his cases the hemorrhage had been alarming.—Dr. Stack advocated using the drug in typhoid cases.—Dr. Edgeworth spoke of the great value of the drug in some cases of enlarged prostate. By its aid the beginning of catheter-life might be postponed.—Dr. Symes replied that he had given urotropine in a score of cases and had not seen hæmaturia produced. It was not broken up in urine which escaped from the kidneys in an alkaline condition.

Dr. D. S. Davies read notes on the case of a seaman who had **variola nigra.** (*See* p. 49.)

---

*February 12th, 1902.*

Dr. Barclay J. Baron, President, in the Chair.

Dr. G. Parker showed a case of **chromidrosis.**—Dr. Stack said they must have admitted six or seven cases like the one shown into the Infirmary in recent years. The blue discolouration had only been between the toes, and had disappeared after maintaining cleanliness for some time.—Dr. Ogilvy alluded to a case he had seen in Dublin, shown and described by Dr. Foote, in which the discolouration was around the eyes and gave the patient the appearance of having spectacles on. This pigment could be rubbed off, leaving white skin beneath, but returned again in about half an hour.—Dr. Parker replied that he had tried to remove the blue stains between the toes in this case with chloroform, ether, soap, paraffin, and other things, but had failed. Wearing white socks had not changed it.

Dr. A. Ogilvy showed a case of **double macular coloboma with persistent hyaloid arteries.** He thought no other case had been described in which the artery persisted in part in both eyes. The coloboma much impaired the vision of the right eye; but the patient could read, having educated another part of the retina.—Mr. C. H. Walker said the case was a very rare one. He thought the persistent portion of the arteries was not more than a few millimetres long. He agreed that the other patch was a congenital defect. Such patches were nearly always met with in myopic eyes.

Dr. J. Odery Symes showed two brothers suffering from **idiopathic muscular atrophy.** In both the wasting had begun at ten years of age.

Mr. W. H. Harsant showed a specimen of **otitic cerebellar abscess.** The patient had been ill three weeks when the speaker first saw him in consultation. He had had occipital headache of severe type on one side, and was not said to have ear disease. On enquiry and examination, however, there was found to be a distinct history of otitis three years previously and perforation of the membrana tympani. He was admitted to the Infirmary. Slow cerebration with dulness and apathy was noticeable. The boy would lie quiet all day and resent disturbance, and did not want food. The speaker had not been able to decide whether the brain abscess he expected to exist was in the cerebrum or cerebellum, and had put off the operation for a day, which he now much regretted, for the next day the boy died quite suddenly. There had been no paresis nor optic neuritis, and no elevation of the temperature, and the pulse had been about normal. Giddiness had not been tested by getting the patient up, owing to his weakness.—Dr. EDGEWORTH thought the reason there were no localising symptoms was that the abscess, as the specimen showed, was in the posterior part of the cerebellum.—Mr. J. LACY FIRTH said he had to regret having some years ago overlooked the existence of a cerebellar abscess, which had also suddenly killed the patient. The patient had been treated for lateral sinus thrombosis with signs of pyæmic abscesses in the lungs, including fœtid and hemorrhagic expectoration, by the radical mastoid operation, obliteration of the left sigmoid sinus and tying the jugular vein. The patient gradually improved, until at the end of six weeks he was getting about the ward and was considered convalescent. After being up a few days, he died suddenly. Reflection after the event led the speaker to think he had perhaps mistaken sluggish cerebration for deafness.—Mr. HARSANT stated his doubt had been as to the cerebellar or cerebral position of the abscess. Cholesteatomatous material had been found in the temporal bone.

Mr. PAUL BUSH showed a **gangrenous ovary (due to torsion) and fimbriated extremity of a Fallopian tube** he had removed from an inguinal sac in an infant, six months of age, with symptoms of strangulated hernia. The child had been ill four days. There was left iliac pain and a swollen left labium, the appearances being those of an ordinary strangulated hernia. The patient recovered. —Mr. ROGER WILLIAMS remarked that the presence of an ovary in the sac of a hernia was not so rare as had been supposed. Many of such hernias were of the congenital type, and due to developmental errors especially affecting the formation of the round and ovarian ligaments. In several cases testicles had been mistaken for ovaries in these cases; and in these cases the external organs of generation had been rudimentary, and the patients had on that account been mistaken for females. Microscopic examination should be made in such cases to settle the nature of the removed body. — Dr. W. SWAYNE considered there was no possibility of the present body being anything but an ovary. The fimbriated end of the tube was quite distinct.—Mr. Bush replied to the same effect, and said that the external generative organs were those of a female.

Dr. G. Parker showed a specimen of **tumour of the brain** and detailed the symptoms in the case.

Dr. W. H. Groves read a paper on a case of **uterine fibroids in which the uterus had been removed two years after double ovariotomy.** Points of special interest in the case were that twelve months after the oöphorectomy and removal of the Fallopian tubes, during which period there had been complete amenorrhœa, the catamenia came on again and recurred at intervals of two or three weeks, and that the fibroid increased in size. The uterus was removed by the abdominal route, supravaginally, and with intra-peritoneal treatment of the stump. A good recovery followed. The speaker discussed the results of oöphorectomy and hysterectomy in the treatment of uterine fibroids. He alluded to some published results in which fifty cases had been followed up after oöphorectomy. In eighteen of these the fibroids had not been benefited. The operation had been largely given up in favour of hysterectomy, owing to the uncertain results of the former and the greatly increased safety of the latter operation due to improved technique.—Mr. Roger Williams said that the operation of oöphorectomy for the disease in question originated from misconception. It illustrated to what a degree second-hand ideas existed in the profession. The procedure was based on the supposed beneficial effects of the menopause. Lawson Tait, finding the results of removing the ovaries only were very uncertain, began to remove the tubes also, and then obtained better results, and so came to attribute the beneficial effects to removal of the tubes rather than to removal of the ovaries. Probably the benefits gained by these operations were attributable to diminution of the vascular supply of the tumours.—Dr. Walter Swayne stated that for the last ten years he had watched several cases of myomata in women who passed the menopause, but in none of these had he found the tumour diminished in size. Ligature of the uterine arteries had benefited some recorded cases. From the specimen, he thought the uterus might have been removed by the vaginal route.—Dr. Groves, in replying, said that he had expected to find oöphorectomy more strongly defended in the debate. In 1897 it had been estimated that 80 per cent. of the cases operated on were benefited by oöphorectomy. He was satisfied that the ovaries had been completely removed in his case.

Dr. Bertram Rogers showed a specimen of **cirrhosis of the liver,** removed from the body of a child, and read the clinical and *post-mortem* notes of the case. The child had for a long period been given beer to drink by his parents. The cirrhosis was considered to be alcoholic. Shortly before death a large collection of ascitic fluid (119 oz.) was removed by tapping. The child died after a copious hæmatemesis. The liver had become firmly adherent to the diaphgram. Its surface was markedly lobulated.—Dr. THEODORE FISHER had regarded the case as one of syphilitic cirrhosis of the liver.—Dr. STACK, from inspection of the specimen, agreed with the view that the liver was a syphilitic one. He had collected thirty cases of cirrhosis of the liver in children for his thesis some time ago. Those due to alcohol were of small, even type. Beer had never produced it, but whisky and gin had.

J. LACY FIRTH,
*Reporter.*

J. PAUL BUSH,
*Hon. Sec.*

---

